# Understanding HIV care delays in the US South and the role of the social-level in HIV care engagement/retention: a qualitative study

**DOI:** 10.1186/1475-9276-13-28

**Published:** 2014-04-08

**Authors:** Courtenay Sprague, Sara E Simon

**Affiliations:** 1 Department of Conflict Resolution, Human Security & Global Governance; and College of Nursing and Health Sciences, University of Massachusetts Boston, 100 Morrissey Blvd, Boston, MA 02125, USA; 2 Wits Health Consortium Faculty of Health Sciences, University of the Witwatersand, No. 8 Blackwood Ave, Parktown (Johannesburg) 2193, South Africa; 3 Department of Conflict Resolution, Human Security & Global Governance, Center for Peace, Democracy and Development, University of Massachusetts Boston, 100 Morrissey Blvd, Boston, MA 02125, USA

## Abstract

**Introduction:**

In a significant geographical shift in the distribution of HIV infection, the US South - comprising 17 states - now has the greatest number of adults and adolescents with HIV (PLHIV) in the nation. More than 60% of PLHIV are not in HIV care in Alabama and Mississippi, contrasted with a national figure of 25%. Poorer HIV outcomes raise concerns about HIV-related inequities for southern PLHIV, which warrant further study. This qualitative study sought to understand experiences of low-income PLHIV on the AIDS Drug Assistance Program in engagement and retention in continuous HIV care in two sites in Alabama.

**Methods:**

The study was designed using grounded theory. Semi-structured interviews with 25 PLHIV explored experiences with care linkage, reported factors and behaviors affecting engagement/retention in continuous HIV care, including socio-economic factors. To triangulate sources, 25 additional interviews were conducted with health and social service providers from the same clinics and AIDS Service Organizations where clients obtained services. Across the narratives, we used the HIV care continuum to map where care delays and drop out occurred. Using open coding, constant comparison and iterative data collection and analysis, we constructed a conceptual model illustrating how participants described their path to HIV care engagement and retention.

**Results:**

Most respondents reported delayed HIV care, describing concentric factors: psychological distress, fear, lack of information, substance use, incarceration, lack of food, transport and housing. Stark health system drop out occurred immediately after receipt of HIV test results, with ART initiation generally occurring when individuals became ill. Findings highlight these enablers to care: Alabama's 'social infrastructure'; 'twinning' medical with social services, 'social enablers' who actively link PLHIV to care; and 'enabling spaces' that break down PLHIV isolation, facilitating HIV care linkage/retention.

**Conclusions:**

Ryan White-funded programs, together with housing, food and psychological support were pre-conditions for participants' entry and retention in HIV care. The path to achieving continuous HIV care for individuals at risk of lack of entry or delayed HIV care requires robust social-level responses, like in Alabama, that address physical and mental health of clients and directly engage the particular social and economic contexts and vulnerabilities of southern PLHIV.

## Introduction

Globally, bold strides have been made to expand antiretroviral therapy (ART) coverage to people with HIV and to arrest AIDS deaths. Eight million people were on ART worldwide by 2012, with sub-Saharan African countries cutting HIV-associated deaths by 30% over the past six years [1]. What’s more, these health gains have been made primarily in the high HIV prevalence, low- and middle-income countries with the greatest burden of HIV infection [2,3].

In the United States, however, gains on the HIV front have stalled. For a high-income setting with sufficient physical and health infrastructures, HIV outcomes remain surprisingly poor. For the 1.2 million Americans living with HIV, just 51% who enter HIV care are retained in care; and only an estimated 25% of those who initiate ART achieve the viral suppression that underpins optimal health [4-6]. Moreover, there has been a marked geographical shift in the US epidemic and its distribution: the South – comprising 17 states – now has the greatest number of adults and adolescents with HIV in the nation [7,8]. By 2010, the southern states captured 45% of new AIDS diagnoses and 48% of AIDS deaths [9].

While survival times and quality of life for people with HIV (PLHIV) have improved dramatically since the introduction of ART, reductions in morbidity and mortality depend on timely HIV care linkage and retention [10-15]. Yet in the South, delayed entry into HIV care appears to be a common phenomenon [16-18]. An estimated 61% of PLHIV in Alabama, and 62% in Mississippi, for instance, are not in HIV care [19,20]. In contrast, an estimated 36% are not in HIV care in the Washington DC/Baltimore metro area [21]. The figure is 34% in New York State [22]. The national figure for those not in HIV care is an estimated 25% [23]. Thus the trend emerging from the data is a lower percentage of southerners with HIV who are linking to HIV care, compared to PLHIV in other regions.

Within the US, the South is distinctive. Characterized by its generally conservative politics, the region has a deep religious base, poorer health infrastructures, lower rates of health spending and tax bases, with longer distances to health facilities than other regions [24,25]. Home to a disproportionate share of low-income residents who are unemployed, many southerners lack access to health care and health insurance. Southern residents have poorer health status, generally, than those in other regions of the US. Southerners with HIV are also estimated to be at higher risk of dying, compared to PLHIV in other geographic locations [26-28].

In recognition of such health and HIV-associated inequities, or differences that are avoidable, unjust and associated with social disadvantage, the US established its first national HIV strategy in 2010, with three primary goals: 1) reducing the number of people who become infected with HIV; 2) increasing access to care and improving health outcomes for PLHIV; and, 3) reducing HIV-related health disparities [29]. Yet the CDC acknowledged that national efforts thus far have failed to reach sufficient PLHIV through testing, ongoing medical care and ART adherence [6]. Research indicates that identifying patients who are at high risk for lack of HIV care linkage, as well as understanding their health behavior related to care entry and retention, is critical. Researchers have also called for use of the growing evidence base to target appropriate HIV interventions to specific sub-populations for improved health and HIV outcomes [27-31].

The rationale and study aim are as follows: given gains made in lower-resource settings and the directives of national public health and HIV goals, the larger health equity questions raised by current US trends are: How do we identify, reach and retain individuals with HIV in continuous HIV care more effectively in the South where new infections are increasing, particularly low-income or socially marginalized populations? [32].

HIV positive Americans with low incomes are able to access expensive antiretrovirals through the AIDS Drug Assistance Program (ADAP). Funded under Part B of the Ryan White HIV/AIDS Program, ADAPs are essentially grants awarded to each state by the federal government to offer treatment to those PLHIV with little or no coverage from private or third party insurance [33,34]. ADAP clients share common characteristics. They are mainly uninsured (72%), male (77%), ethnic minority groups (63%), with incomes at or below 200% of the federal poverty level (75%). Over half (51%) will initiate treatment with CD4 cell counts of 350 or below [35]. Lower CD4 cell counts are sub-optimal for achieving viral suppression, with greater risk of morbidity and mortality [36,37]. Enrollees on the AIDS Drug Assistance Program thus offer an important window into understanding low-income PLHIV in the US with high-risk behaviors, who may also have co-morbid conditions and experience health inequities. Yet our understanding of ADAP clients and the social and structural factors that affect their HIV care linkage and retention is limited [38-40].

To date, few qualitative studies have focused on HIV care and retention for socially marginalized groups in the southern US, likely due to an historical concentration of HIV in pockets of the Northeast, Mid-Atlantic and West [28]. ADAP studies have emphasized clinical outcomes, eligibility, financing, equity and cost effectiveness [41-45]. No qualitative research was found, however, to explore links between ADAP use in the South and entry, linkage or retention in HIV care for low-income PLHIV and underlying social factors and determinants of health. Yet the complexity of individual-, social- and structural-level factors interacting in linkage to care for vulnerable groups lends itself well to qualitative methods [46-49].

In response to this gap in the knowledge base, the aim of this qualitative study was to understand experiences of low-income PLHIV on the AIDS Drug Assistance Program (ADAP) in linking to, and retention in, continuous HIV care in two sites in Alabama. The research question was exploratory: *For people living with HIV (PLHIV) on the AIDS Drug Assistance Program in the US South, what is their experience accessing the continuum of HIV services, including linkage and retention, and managing or promoting their health within their socio-economic contexts?*

## Methods

Transcriptions, self-reported information (number of years living with HIV, CD4 cell counts, viral loads, history of substance use and incarceration) and social-demographic data comprised the primary data. The authors used grounded theory in the qualitative study design, subsequent data collection and analyses to generate an “explanation of social phenomena under study” [50,51]. The stages of data collection and analysis employed were: open coding to create concepts that emerged from participant descriptions; constant comparison of concepts to identify main categories (themes) that emerged, while conducting further iterative data collection and analysis to probe and verify themes. A semi-structured questionnaire was utilized to engage respondents in detailed conversations about their experiences with health and HIV care and health behaviors, their social and economic contexts and related factors. These face to face interviews with 25 PLHIV respondents allowed for collecting thick and rich descriptions of individual experiences with care and to probe linkage, lapses in care (drop out), as well as reported barriers and factors affecting HIV retention in long-term continuous care.

In terms of research settings, Alabama was selected as a southern state with rising HIV incidence, a significant percentage of individuals in the state in poverty - 54% of the state’s population is living 300% below the federal poverty level - with poor health status for residents generally, high reported need for HIV care linkage and high utilization of the AIDS Drug Assistance Program by state residents [52]. With a population of 4,785,401, Alabama had an estimated 10,462 people with HIV in 2008 [53]. In 2010, ADAP served 2,070 HIV positive clients in Alabama [54].

Two locations were chosen as research sites: Birmingham (south central Alabama) and Tuscaloosa (west central). Sites were identified on the basis of having a health clinic with an AIDS Service Organization (ASO) nearby: i.e., individuals would access health care from their clinic and obtain social services from their local ASO. The clinic and ASO thus served the same PLHIV population, with a relationship of referral and information sharing: allowing investigators to explore PLHIV use of both social and medical services in context.

The two clinics served a large share of low-income clients, with a considerable ADAP client pool and documented rises in new HIV patients. For example, the Birmingham clinic’s PLHIV patient load increased from 500 PLHIV patients in 1997 to 2,000 PLHIV patients in 2012. The Tuscaloosa facility had 319 PLHIV patients in 2012: with 39 new ones in the first 10 months of that year alone (interviews with key informants, 5-9 November 2012).

The PLHIV sampling strategy was to select a diverse, information-rich pool of participants with a range of experiences: those newly diagnosed, for instance, and those living with HIV for many years. Respondents who qualified for, or were already accessing ART through the AIDS Drug Assistance Program, were recruited. Participants were sought from all ethnic and educational backgrounds, male and female, heterosexual, gay and bisexual. Characteristics of the study population are summarized in Tables 1 and 2. In Birmingham a total of 11 PLHIV participants were interviewed. In Tuscaloosa, 14 PLHIV participants were interviewed, or 25 PLHIV.

**Table 1:** Characteristics of study population: age, education, employment, race, sexual orientation and gender

**Characteristics**								
**Age**	20-52		**Education range**		7th grade to associates degree	**Unemployed**		14
		**Race**				**Sexual orientation**		
**Sex**		African- American	White	Multi-racial	Hispanic/Latino	Hetero-sexual	Bi-sexual	MSM
**Male**	18	13	4	1	0	6	4	8
**Female**	7	3	3	--	1	7	--	--
**Total**	25	16	7	1	1	13	4	8

**Table 2:** Years living with HIV, mental health, substance use, food, housing, transportation challenges, previous incarceration

**Years Living with HIV (as of November 2012)**			<1 to 24 years			
**Number reporting mental health challenges, substance use, food, housing and transportation challenges and previous incarceration, of 25 people total**						
	Mental Health Challenges (including anxiety, depression, bipolar disorder and suicidal thoughts)	Previous or current users of substances (some currently in rehabilitation programs)	Actively using food stamps or expressed challenges accessing food	Difficulty with housing or homelessness	Previous incarceration	Use ASO transportation vouchers, food and/or rehab programs
**Number self- reporting**	16	9	19	5	7	25

To triangulate data sources, we sought the perspectives of health providers and ASO staff, purposively selecting those who engaged directly with PLHIV clients in the two neighboring clinics and two ASOs, to further understand care linkage and retention for the PLHIV in the study sample. The first group comprised *health providers*: nurses, doctors and medical social workers who cared for individuals on ADAP. The second group encompassed *social service providers* who staffed ASO prevention, housing or transportation programs. We interviewed 25 key informants, bringing the interviews to 50 total.

CS conducted the 25 PLHIV interviews and coded, while SES independently coded. SES and CS conducted in person and telephonic interviews with the 25 key informants (health and social service providers). Working through the data collection cycles, we identified common concepts, categories and themes. We deployed the process of cotemporaneous data collection and analysis of grounded theory to understand and generate a model to represent how linkage to HIV care and retention occurred for study participants in the two sites in Alabama.

Formal IRB approval was received from the University of Massachusetts Boston (2012126) and the University of Alabama Birmingham (X121003004). The study purpose was explained, and only participants who gave informed consent were interviewed. PLHIV were given a stipend of $25 each to accommodate travel and time.

**Figure 1:**
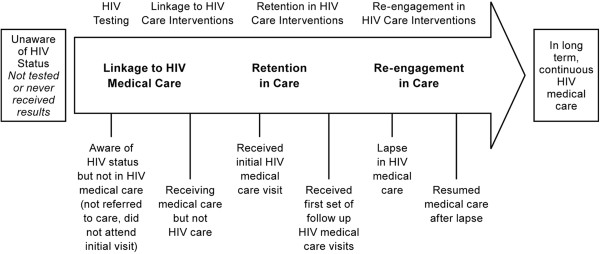
**HIV care continuum. **Sources: Adapted from Health Resources and Services Administration, HIV/AIDS Bureau, 2006, p. 4; and CDC, 2013; Available from: http://www.cdc.gov/hiv/prevention/programs/pwp/linkage.html.

Conceptually, we used the HIV care continuum shown (Figure 1), alongside the narrative reports, to visually identify and map patterns in engagement or care delays, with a view to pinpointing where delays, lapses and drop out occur. HIV care is a process where an individual begins to engage in a care ‘continuum’ through HIV testing and diagnosis, routine monitoring, and ART. The initial point of linking a patient to HIV care is called entry, engagement or linkage, though common definitions and use in the literature are far from uniform [55-57]. Retention refers to maintaining patients in HIV medical care for routine medical visits over time [58-60].

The care continuum is a visual representation of a spectrum of HIV care for an individual. At one extreme, the left-hand side of the continuum is the individual who is not in HIV care. At the other, moving to the far right-hand side of the arrow is the individual fully retained in care (the ideal) [61,62]. Lapses may occur and individuals then need to be ‘re-engaged’ in care. The end goal is to retain the individual in continuous HIV care to achieve optimal health outcomes, including suppressed viral load and reduced risk of illness and premature death.

In the next section we present the study findings, locating these within the HIV care continuum. In the discussion section, we then generate a new conceptual model to capture how respondents described their paths to HIV care entry and retention.

### Findings

1. Delayed Care: First Experience of HIV Testing (on Diagnosis) and Respondents’ Entry or Failure to Link to HIV Care

Almost all 25 PLHIV respondents spoke of their experience of HIV testing as a singular event, recalling how they felt at that instant of receiving positive results and their corresponding actions - to pursue treatment or not. The first finding was one of delayed care and the impact of HIV test results affecting entry or lack of linkage to HIV care. A majority of respondents shared their histories of substance use, indicating that following receipt of a positive diagnosis, they were vulnerable to returning to self-harm behaviors, as illustrated in the following narratives.

### Return to substance use

A 48 year old African-American heterosexual male and previous substance user diagnosed roughly two years ago, described himself as being “stunned.” He said, “It was like time stopped and his mouth [the counselor’s] was moving but I could no longer hear what he was saying.” He said: “I had suicidal thoughts.” He asked himself, “Where did I get it? How do people view me? …” He said he immediately went back to using drugs [without linking to HIV care]: “I wanted to give up on life… I was punishing myself. I used alcohol, cocaine…” (patient 11, Birmingham).

A similar pattern surfaced, with other respondents “overwhelmed” on learning their status. One respondent said: “I went back to drugs” (patient 2, Birmingham). A second stated: “I went on a bender” (patient 4, Birmingham). A third, currently in a drug rehabilitation program, indicated on learning her HIV positive status: “I fell apart” (patient 12, Birmingham).

**Figure 2:**
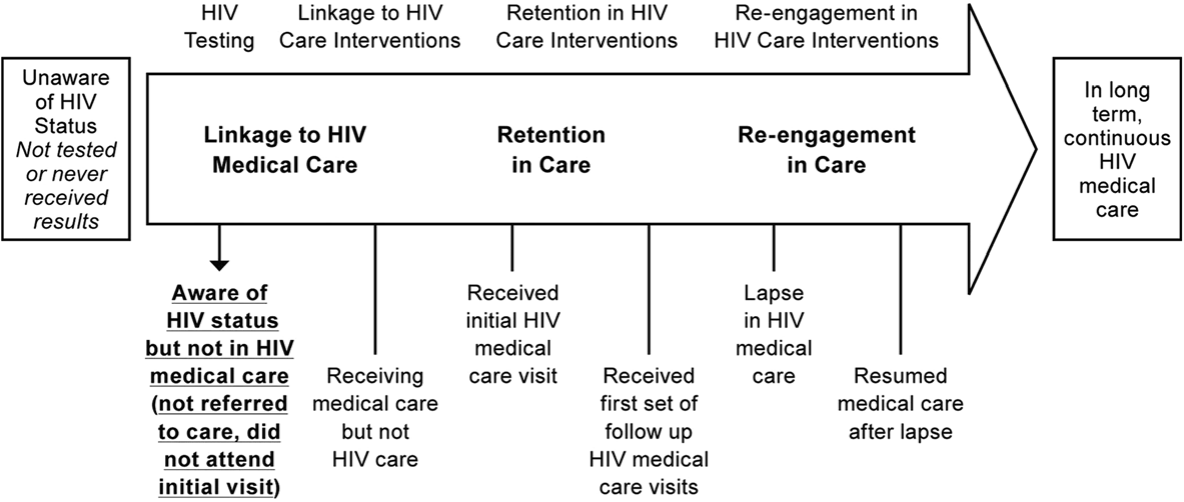
Aware of HIV status but not in HIV medical care.

Significantly, as indicated in bold in Figure 2, though these individuals were aware of their HIV status, many did not advance to the next stage of the continuum: they failed to link to HIV care and to initiate antiretroviral therapy, some for a number of years. This became a visible point of drop out of the health system.

2. Treatment Delays – Only Initiating ART When Ill

In addition to a return to substance use, the second finding was one of respondents’ delaying their care and treatment until ill in the hospital. The following accounts capture this pattern.

A 47 year old white woman reported “a horrible experience” being tested for HIV in 1992. Her doctor said she probably had “two years to live.” She said, “I got hysterical and left.” After five years without taking HIV medications, she developed pneumonia, prompting her to begin treatment. She said, “I thought if I want to live, I better start taking some meds” (patient 8, Tuscaloosa).

A 51 year old African-American heterosexual man donated blood for the Red Cross in 1988, learning he was HIV positive during the screening process. He did not access HIV medications for six years. He recounted: “Once I had gotten really sick, my CD4 dropped to four. I was hospitalized with pneumonia.” The doctor encouraged him to get onto treatment if he wanted to get well and stay alive. After he was released from hospital, he started AZT, and later dual and triple combination therapy (patient 12, Tuscaloosa).

A Latina woman aged 43, living with HIV for 23 years, a former user of substances who was previously incarcerated, did not link to HIV care until she had been admitted to hospital for pneumonia four times. She said it was the doctor’s question that finally prompted her treatment initiation nine years after she was first diagnosed. She recalled: “In the hospital, the doctor asked me: ‘Are you ready to meet your maker?’ and it dawned on me to start taking medication or die” (patient 3, Birmingham).

**Figure 3:**
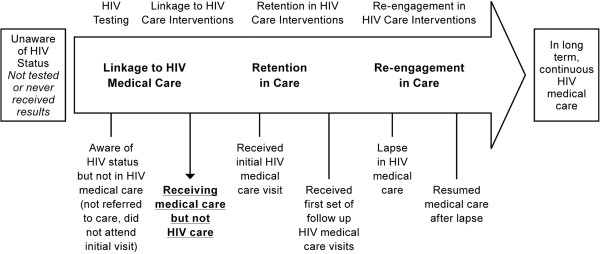
Aware of HIV status and in medical care but not HIV care.

As indicated in Figure 3, though these individuals eventually accessed medical care for reasons of ill health, many faced barriers to HIV care engagement, including during incarceration.

3. Incarcerated Sub-Population Lacking Linkage to HIV Care in Prison and ART Lapses/Drop off

The third finding was specific to individuals who had been incarcerated: Seven formerly incarcerated respondents shared experiences of inadequate linkage to care while in prison, with HIV testing and no counseling, treatment interruption and lack of access to HIV medication.

Respondent 3 (Birmingham), a heterosexual Latina woman, 43, with previous use of drugs, was on ART but denied medication for 5.5 months while incarcerated in the 1990s. She spoke of being segregated as an HIV positive prisoner, wearing a white armband to denote her status and being denied food. She indicated that “AIDS” was written in marker on both sides of her uniform basket.

Respondent 7 (Tuscaloosa), a 38-year old African-American heterosexual male was incarcerated in the county jail for 10 months and later moved to Limestone Correctional Facility for five. Limestone is a maximum security prison (near Huntsville in north central Alabama) where all male HIV positive inmates were held until 2012 [63]. He said: “They segregated the prisoners.” “HIV positive people could only be in certain places [in prison] – we had to eat with HIV positive prisoners only.” He was unsure of his viral load and CD4 cell count during that period and never got onto treatment while incarcerated. After he was released, he initiated ART.

Respondent 8 (Birmingham), an African-American gay man, aged 34 with a history of substance use, was incarcerated for three years. He spoke of being segregated in a designated dorm due to his HIV status, and wearing the white armband. Describing his experience as “horrible,” he said he was “grateful” to get his medications eventually “because people from 1917 clinic [doctors from Birmingham] fought for our rights in 2008,” ensuring their access to ART.

Interviews with key informants and published reports confirmed that male inmates were segregated based on their HIV positive status, in keeping with state prison policies in Alabama until December of 2012, when a court judgment overturned these practices [64]. With an estimated 265 HIV positive male and female inmates in Alabama prisons, the full spectrum of physical and mental health needs of inmates is unknown [65].

**Figure 4:**
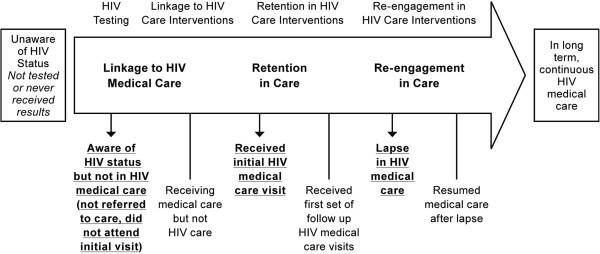
No treatment or treatment interrupted/denied.

As shown in Figure 4, individuals who experienced periods of incarceration either lapsed in HIV treatment because they were not able to access ART in prison; or they were never initiated. This was in spite of the fact that their HIV positive status was known to prison authorities, who moved inmates to Limestone and segregated them in the facility because of their HIV serostatus.

4. Diverse Conceptions of What PLHIV Need to be Healthy and Engage/Re- Engage in Care

The fourth finding stemmed from two open-ended questions asked of respondents, prompting their reflection and discussion relating to their linkage to and retention in care: 1. What are the challenges you face in managing your health and HIV status? 2. What do you need to be healthy and stay in HIV care? This finding captures the span of respondent perceptions. The first concerns the need for stable housing, which is closely followed by and linked to the need to secure transportation and adequate food. Support, rehabilitation, education, employment and spiritual needs are also identified.

### Housing

Half of the respondents mentioned housing as important to them to be healthy. Respondent 7 said he needed housing, medical care and food. He stressed that he must focus on his spiritual needs through church and prayer. He relied on “doctor visits and groups such as Lunch and Learn” [weekly educational program at the clinic about various aspects of HIV]. He also emphasized his need for mental health support, employment and insurance (Birmingham).

Respondent 6 said he was struggling “with rehab,” and getting his appetite back. He needed more information about HIV, stating he didn’t know much about it. He stressed that he needed “housing stability” (Birmingham).

Another individual hospitalized for a blood clot noted, when he was released, he became “kind of homeless.” He moved in with his mom and said: “She had an abusive boyfriend who jumped me.” He then entered the AIDS Alabama emergency bed program,” which he described as a gateway into HIV care and greater stability. He repeatedly mentioned his need for “housing to get my life back on track” (patient 10, Birmingham).

Similarly, respondent 3 stressed: “Your own housing gives you freedom and control… health providers you can call…” faith as “foundation and security” and “people and friends to support you”…(Birmingham). Respondent 5 emphasized that he needed to “stay clean and sober.” He emphasized: “housing, employment…” as important for his heath; and “for my CD4 counts to come up” (Birmingham).

### Transport

Another dominant need revolved around transport. More than one third of respondents talked about challenges in accessing transportation. Transport was mentioned, not only in conjunction with attending medical appointments but also getting to work and shopping for food. A 45 year old African-American woman expressed concern about getting to her job, which was far away and very expensive by taxi. She could walk to the clinic for her medical appointments. But she mentioned wanting a car to simply go home rather than waiting an hour for the bus. She said she would likely be too tired to fix something to eat by the time she returned home (patient 14, Tuscaloosa).

### Food

Nineteen of the 25 PLHIV interviewed expressed challenges accessing adequate food. Patient three, a Latina heterosexual female aged 43, said she had to choose what to buy: “food or other things” (Birmingham). Patient six (Birmingham), a 28 year old gay white man commented that he gets food stamps but runs out of them and needs more money for food assistance. Patient 14, age 45, an African-American heterosexual woman stated, it is “hard to save enough to get food” (Tuscaloosa).

**Figure 5:**
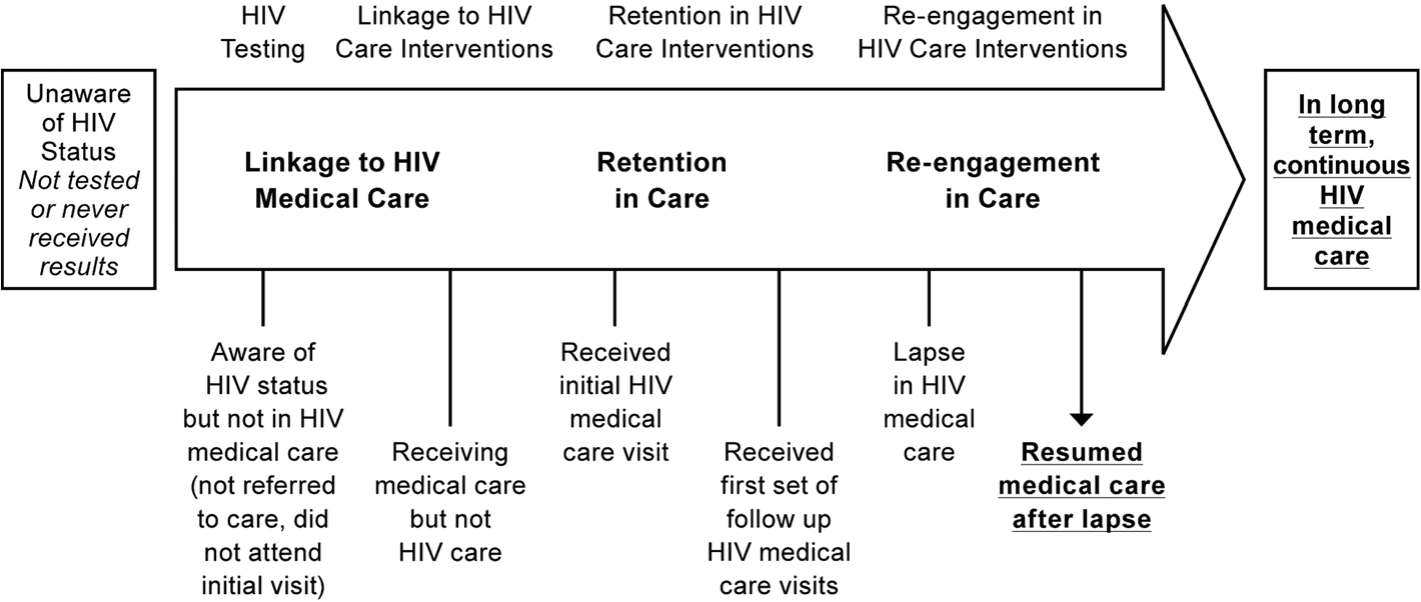
Re-engagement and retention in continuous hiv medical care.

These narratives reflect the overlapping circles of physical, spiritual, mental health, economic and social services individuals reported needing to access, in order to engage or restart long term continuous HIV care, following delayed care or drop out, as shown in Figure 5.

## Discussion

Not pre-selected during recruitment but emerging from our sample was a visible set of risk types shared by respondents to a greater and lesser extent. Types of risk could also be described as overlapping vulnerabilities. Individuals were not just economically-poorer, they experienced concentric mental health challenges and behaviors that increased their risk of acquiring or transmitting HIV, including substance use, periods of incarceration, together with high risk (anal penetrative) sex. One key informant, a social worker at an ASO in Birmingham, referred to clients as often presenting a “triangle” of characteristics - “mental health issues, substance use and incarceration” (18 March 2013).

Theme 1. Shared experiences of delayed care

The care continuum allowed us to map and identify patterns in HIV care linkage and retention. In pinpointing where individuals engaged, lapsed, or failed to go further, we saw a first prominent theme among these respondents: a clear pattern of delayed care, which was also not a recruitment criterion, but a common factor that became apparent. Individual accounts, presented in the findings section, revealed both the challenges of linking to HIV care following diagnosis, as well as fears of and/or lack of information about starting ART. The interviewees communicated factors that led, and sometimes drove, people into HIV care: a social worker’s words of advice or a doctor’s frank prognosis.

The question that emerged during our study was: how do people eventually link to care? All people were in HIV care at the time of the interview (a recruitment criterion, which we verified in interviews). In describing how they began accessing HIV treatment following periods of delay or lapses, we saw convergence around a set of themes that allowed us to understand how respondents experienced care; and, for many, went from being out of care (generally delaying care until ill) to being in continuous HIV care. Based on the narrative reports in interviews, and through the iterative process of data collection and analysis associated with grounded theory, we generated a model to represent this. First we elaborate the additional primary themes that emerged; then we present the model.

Theme 2. Structured social services: a social infrastructure in Alabama to link, re-engage and support PLHIV in care

The large reach and ubiquity of these ASO programs became visible. They played a linking role to the clinic, but as important, they responded directly to the needs of these PLHIV in their social and economic context. What came to light as a second clear theme was the “social infrastructure” to support PLHIV in the Alabama sites, which could be instructive for other states, settings and countries. The two ASOs had programs for housing (owning units that could be let to PLHIV tenants), food and rehabilitation. They had budgets to cover limited utility bills for PLHIV and offered ‘care’ packages with essential supplies: soap, toothbrushes, toothpaste and laundry detergent. Ten ASOs covered 67 counties in the state. While these are located mainly in urban areas, efforts were made to reach clients one to two hours outside the two cities, with indications that other Alabama ASOs make similar efforts (interviews with key informants, 5–9 November 2012). The public transit system in these two cities was described by participants and key informants as insufficient, with some areas of the city inaccessible by bus, and several hour delays for buses to frequent other locations in the city.

*“Twinning”* Significantly, the two ASOs had relationships with their neighboring clinics and served the same PLHIV. The ASOs are community-based organizations (CBOs) that appeared to reinforce and act to “twin” social services with the medical services individuals received in clinics. What emerged was a picture of the social process for PLHIV in Alabama to link to HIV care, and their reliance on CBOs as a vehicle for securing the types of Ryan White and other social programs that allowed them to find needed stability in their lives. Inside the clinics, the medical social workers played a key role in this “social infrastructure” too.

Theme 3. Social enablers: social workers inside clinics and ASOs play active role in linking, re-engaging and retaining PLHIV in care

A third theme that became evident from individual narratives: when individuals linked to care they did so through the active agency of a person - a social worker in the ASO or health clinic. Alternatively, a doctor telling them that ‘time was ticking’ and they needed to start ART, with individuals finally ready to do so. We refer to these actors who engaged PLHIV in understanding what they needed to do and where to go, thus facilitating a path to care, as ‘social enablers’.

In particular, an important nexus was observed between social workers and patients bound together by the ADAP and related paperwork that low-income patients would complete to access various Ryan White social programs [66]. Social workers described how, concerned for patients with low CD4 cell counts, they act rapidly to start patients on therapy, employing short-term compassionate (drug) use programs. They then switch patients onto ADAP for the long term. From interviews with the social workers, it became evident that this requires a set of skillful gymnastics, as they negotiate qualification criteria and accompanying reams of bureaucratic paperwork to initiate patients onto ART (key informant interviews, 5–8 November 2012).

Theme 4. Enabling spaces

The fourth theme was one of ASOs creating ‘enabling spaces’ for people in Alabama to learn about resources, programs and HIV support: all essential steps to link to care and to feel empowered to do so. Very noticeable was the lack of HIV information in the two cities (outside of the CBOs and clinics), an observation confirmed by key informants. Key informants reported that HIV is not part of the educational curriculum in schools across the state, with no health campaign to sensitize the public about HIV. Given the rising numbers of HIV infections in the South, this has negative implications for the health of southerners with HIV and those at risk of HIV acquisition.

In the United States, individual and social-level effects of HIV are hidden, likely due to the lower prevalence and priority attached to HIV. In contrast, HIV is increasingly recognized as a social epidemic in other parts of the world, such as southern Africa, where adult HIV prevalence in nine high prevalence countries ranges from 11% in Malawi to 26% in Swaziland [67-71]. The practical reality of one in four or five people living with HIV in southern Africa has meant that access to HIV information and awareness is common, as part of broader multi-stakeholder social responses. The social, political, economic and household effects of the epidemic are thus visible and cross-cutting. In locations like South Africa, HIV has had much more transformative social effects as a result [72-74].

What these reports with low-income PLHIV in Alabama indicate is the path to achieving continuous HIV care requires attention to social needs, as well as the agency of individuals, who, in a vacuum of HIV information, offer resources and break down isolation. What is thrown into relief here is the interplay between the individual, the social and the structural, which has been documented in other studies [75-77].

**Figure 6:**
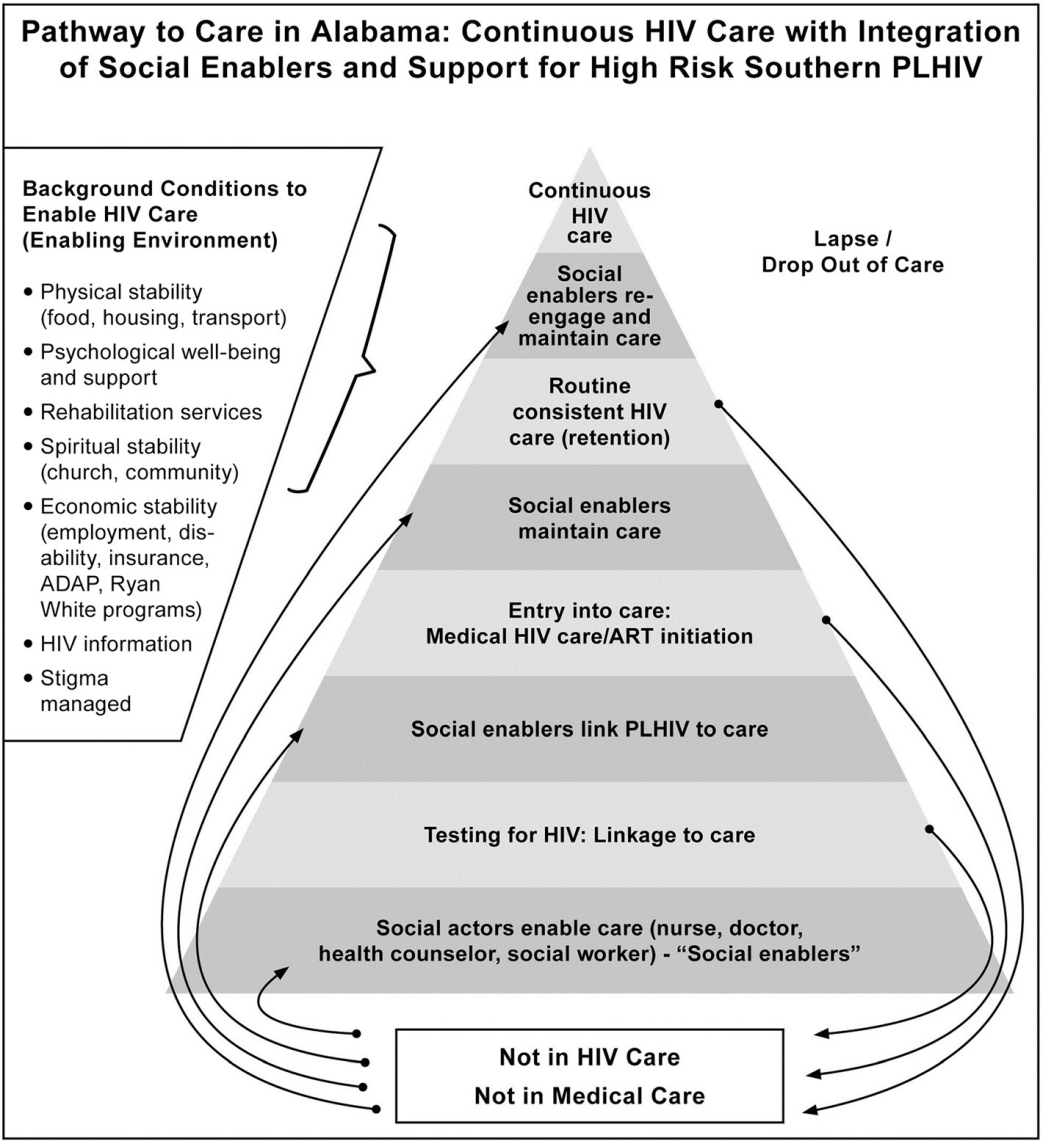
Pathway to care in Alabama: continuous HIV care with integration of social actors and social support for high-risk Southern PLHIV.

Below we present a diagram (Figure 6) to capture the social infrastructure – the services, social enablers and related factors – in the two locations in Alabama – which may not be present in other poorer southern states, such as in Mississippi [78].

In this model, we represent the steps to the goal of continuous, consistent HIV care as a pyramid, with fewer numbers of individuals reaching the summit due to drop off at each stage. The pyramid is located in a larger ‘box’ (shown in figure) to demonstrate that it is framed by an enabling environment. For that background environment to enable HIV care, physical, economic and spiritual needs must be addressed (the extent to which needs are met will vary); HIV information must be made available; and stigma must be managed to some degree. The components of this enabling environment were expressed by respondents, e.g., one where needs for food, transport, housing, insurance, mental health support and rehabilitation were immediate prerequisites to longer-term HIV care. These needs remain conditions to be addressed throughout the care trajectory. Therefore they are represented in the background and not the pyramid itself. Individuals who are not in HIV care, meaning they have never been tested or not linked to care, are outside the box. Individuals who are in medical care (but not HIV care) are also located outside the box.

As an individual progresses in care (demonstrated by moving up the pyramid): from testing to entering HIV care, to remaining in care and achieving consistent, continuous HIV care, it is these social enablers that provide the link to the next step. At each critical stage, it is the support of these individuals that encouraged care linkage (illustrated by the curved arrows). For example, testing and then counseling someone newly diagnosed to engage in care, providing the transport voucher to reach the clinic, providing housing or food supplements to enter or re-engage in care.

The model recognizes drop out and lapses at each stage and that some individuals may and do encounter ‘disablers’ who discourage progression to continuous care, through stigmatizing remarks, for instance. As one PLHIV participant from Birmingham indicated, he switched doctors because “the other doctor wouldn’t touch me. He wouldn’t touch my body” (patient one). And yet, with the background enabling conditions and social enablers, an individual can, equally, make her way back into long-term, continuous HIV care, even if dropout or lapses have occurred.

There are limitations to this study. First, reliance on self-reports may magnify the most immediate health concern on the respondent’s mind on the day of the interview, perhaps minimizing corollary concerns of equal importance over a longer trajectory in their HIV care. Second, this sample of PLHIV may not be representative of all PLHIV in the state or region, though the characteristics of these respondents are shared by PLHIV within Alabama as well as neighboring PLHIV in southern states where care delays have been reported [79,16-20]. Third, we did not differentiate care delays or experiences by sub-population, such as MSM or users of substances, which may be important in yielding further insights.

At present, few qualitative studies have been conducted related to HIV care and retention for socially marginalized groups in the southern US, likely due to the historical concentration of the early HIV epidemic primarily in cities in the West and Northeast. Research in the southern US has thus far focused on depression, substance use, virological failure, viral suppression, care delays and barriers to HIV retention for women. To date, ADAP studies have emphasized clinical outcomes, eligibility, financing, equity and cost effectiveness [38,41-45]. No qualitative research was found, however, to explore links between ADAP use in the South and entry, linkage or retention in HIV care for low-income PLHIV and underlying social factors and determinants of health. Thus, in spite of limitations, this study fills a gap.

The research is important in bringing an applied social science perspective to understand delays in HIV care in the selected sites. This qualitative study achieved greater understanding of the experience of low-income PLHIV in the Alabama sites, offering insight into intertwined social-level factors that affect HIV care linkage and retention, namely: 1) The social process of care and the social infrastructure created in Alabama by ASOs and clinics; 2) The importance of “twinning” social and medical services for high risk southern PLHIV in Alabama which reportedly aided these PLHIV in HIV care linkage and retention by meeting social and medical needs in tandem; and 3) The role of social enablers in actively linking to care populations at high risk for lack of linkage, like many PLHIV in the South, as captured in this new model of care.

Given that over 60% of PLHIV in some southern states are not in HIV care, future research could focus on those PLHIV in the South who have never linked to HIV care [80], including incarcerated populations, as well as challenges to long-term retention for those in HIV care, with studies aimed specifically at those ‘lost to follow up’.

### Recommendations

These selected recommendations are targeted to overcome HIV care delays in the study population by linking PLHIV to care and support more rapidly in Alabama; and retaining them in long-term continuous HIV care.

Recommendation 1: *At key moment of diagnosis, follow up with PLHIV using text messaging, phone calls, community liaisons or peer support to break isolation and establish care linkage*

The first experience of HIV testing (on diagnosis) was a significant moment for these PLHIV, creating a critical opportunity for care linkage – one that appears to be time sensitive. Findings indicated that individuals with patterns of substance use return to them, while individuals with mental health challenges show evidence of being vulnerable to risk and self-harm behaviors. Thus, engaging PLHIV early – to break their isolation and engage them in support - is critical. Timely communication could occur through community-liaisons, which Alabama ASOs have utilized: i.e., an individual who goes into communities to provide counseling and explains the range of HIV and support services available. A social worker or peer could also reach out to PLHIV by text message or phone. Due to the high reported challenges associated with mental health (19 of 25 PLHIV), repeated counseling (rather than ‘once off’) and psycho-social support, are also vital.

Recommendation 2: *Better support and enable social workers to get individuals into HIV care more easily and effectively by reducing ADAP bureaucracy*

Social workers were over-burdened with unnecessary paperwork that took their time away from attending to patients’ health. We recommend simplifying and streamlining the ADAP recertification process. For example, every six months individuals should not need to retest for HIV. States determine eligibility criteria for ADAP and this criterion in Alabama is changeable. Recertification could occur yearly, generating greater efficiency gains and shifting the focus to patient-centered quality of care. In addition, electronic, rather than the current, paper systems, could be put into place to minimize duplicate paperwork and build a state-wide database to better support patients who change clinics or locations.

Recommendation 3: *Secure continued ADAP and Ryan White program funding in Southern States during health care transition*

During the transition to implement the Affordable Care Act, states will make important decisions affecting health care. Significantly, they will decide whether or not to extend Medicaid and determine which health plan will become the benchmark for essential services. For PLHIV, Ryan White programs, including ADAP and related social programs that support their health - housing, substance rehabilitation, food programs - are absolutely critical to achieve the stability in their lives that then allows them to engage in HIV care and move towards continuous long-term retention. Since HIV outcomes in Alabama are currently sub-optimal, with low-income individuals delaying care and at greater risk of dying, removing such programs is likely to produce even poorer health and HIV outcomes. In this time of transition, it is vital that federal funding and Ryan White programs, including ADAP, are continued.

Recommendation 4*: Increase public awareness of HIV in Alabama in order to tackle HIV stigma and offer HIV prevention messaging*

Because of the lack of HIV information and knowledge among the general US population, fear and misinformation can and is directed at people living with HIV. Several actors could play a key role in increasing public HIV awareness. Data on residents in the state of Alabama from this and other research indicate that residents tend to be church-going and religious [81]. Churches can be an important ally in HIV education and could become an additional ‘enabling space’ in Alabama - if they were to create welcoming, supportive environments for parishioners and others with HIV. Pastors, priests and lay people could all play a role in offering outreach and social support. Continuing ASO outreach remains necessary, as these trusted CBOs are able to reach and educate people in locations they naturally frequent: e.g., at the hairdresser or local beer hall. AIDS Alabama maintains significant outreach in these two venues through their programs, “Many Men, Many Voices” and “Beauty and Knowing” (interviews with key informants, 5–9 November 2012). Yet HIV infections are still increasing and greater outreach is needed. Given the lack of public information about HIV transmission and risk behaviors, the Alabama Department of Public Health could invest in outreach and public health campaigns to sensitize people to the modes of HIV transmission, to educate residents and address stigma.

Recommendation 5: *Reach incarcerated populations with HIV services and remove punitive measures that impact access to social programs for former inmates, such as food stamps and housing*

Given Alabama’s decades of segregation of HIV positive inmates, there is scope for ensuring individuals with HIV in prison or jail (and after), who may learn they are HIV positive for the first time while incarcerated, receive timely counseling, treatment and referral information; while also ensuring that stigma and discrimination are not practiced in those settings. Individuals with certain types of convictions are barred from accessing social programs in the state of Alabama, including food stamps (vouchers) and specific housing programs (interviews with key informants, 5–9 November 2012). This research indicates that PLHIV who want to rebuild their lives face multiple barriers and need every available resource at their disposal to manage their health and interrupt the cycle of re-incarceration. Punitive policies only make re-entry into society more difficult, while making the transition to positive health behaviors more challenging.

## Conclusions

Global strides have been made in averting new HIV infections, reducing HIV-associated mortality and scaling ART, particularly in the low and middle-income countries. In the US, improvements have not been made in HIV incidence, HIV retention or viral suppression. In some southern states, upwards of 60% of individuals with HIV are not in HIV care, contrasted with a national figure of 25%. In the South, poorer health status and HIV outcomes are evident, compared to other regions in the United States, creating the rationale for this research.

At the national level, there is greater recognition that such HIV-related health disparities in the US demand new approaches. Indeed, the US National HIV/AIDS Strategy recommends “more comprehensive responses to social service needs” [82].

Given the gains made in lower-resource settings and the directives of national public health and HIV goals, the larger health equity questions raised by current US trends are: How do we identify, reach and retain individuals with HIV in continuous HIV care more effectively in the South, where new infections are increasing, particularly in low-income or socially marginalized populations? The aim of this qualitative study was to understand experiences of low-income PLHIV on the AIDS Drug Assistance Program (ADAP) in linking to, and retention in, continuous HIV care in two sites in Alabama. This research achieved greater understanding of the experience of low-income PLHIV in the Alabama sites and their pathway to care, offering insight into intertwined social-level factors that affect HIV care linkage and retention. This encompasses: a) the social process of care and the social infrastructure created in Alabama by ASOs and clinics; b) the importance of “twinning” social and medical services for high risk southern PLHIV in Alabama, which reportedly aided the respondents in care linkage and retention by meeting social and medical needs in tandem; and c) the role of enabling spaces and social enablers in actively linking to HIV care those populations at high risk for lack of linkage, like many PLHIV in the South, as captured in this new model of care.

Alabama ASOs and health clinics are at the frontlines of the US HIV response. In communities in the South, demand for these services is increasing. If we want to achieve national goals of a seamless health care system that engages ‘hard to reach’ PLHIV, we must work harder to inform our understanding of how to address HIV-related inequities at the social-level [83]. A key challenge is to work with local actors, such as CBOs, to use research to inform action that better engages and retains southerners with HIV in long-term continuous HIV care. As Cheever et al. note: “their lives depend on it” [58].

## Abbreviations

ADAP: AIDS drug assistance program; AIDS: Acquired immune deficiency syndrome; ART: Antiretroviral treatment; ASO: AIDS service organization; CBO: Community based organization; CDC: Centers for disease control and prevention; HIV: Human immunodeficiency virus; MSM: Men who have sex with men; PLHIV: People living with HIV and AIDS; UNAIDS: the Joint United Nations Programme on HIV/AIDS; US: United States of America; WHO: World Health Organization.

## Competing interests

The authors declare that they have no competing interests.

## Authors’ contributions

SES and CS together conceived the study design, execution and coordination, conducted telephonic and in person interviews from May to November 2012, analyzed and interpreted the data, and drafted sections of the manuscript. CS conducted the 25 PLHIV in-person and additional key informant interviews at the two research sites in November 2012. Both authors read and approved the final manuscript.

## Authors’ information

SES is a development policy specialist engaged in HIV work for nine years. She has directly supported civil society and people living with HIV at the UNAIDS Program Coordinating Board for four years, where she focused on understanding barriers PLHIV in different settings experience that may inhibit their access to HIV care, treatment and support, as well as communicating this research and information to the UNAIDS board on behalf of civil society organizations around the world to inform the global response. CS is an applied social scientist engaged in the HIV field for 14 years, based at the University of the Witwatersrand, South Africa for nine years, and now the University of Massachusetts Boston (USA). She seeks to understand fundamental health inequities and to address the needs of those PLHIV with the poorest HIV outcomes - those in lower-resource settings - by exploring the interaction of social and structural factors on health and health achievements, as well as appropriate levers for intervening to improve health – through quality health services’ delivery, reform and appropriate social policies. They have co-published together previously on employment discrimination and HIV stigma. See Sprague, Laurel, Sara Simon and Courtenay Sprague: Employment Discrimination and HIV Stigma: Survey Results from Civil Society Organizations and People Living with HIV in Africa. *African Journal of AIDS Research* 2011, 10(3): 311–324.
